# Small Area and Individual Level Predictors of Physical Activity in Urban Communities: A Multi-Level Study in Stoke on Trent, England

**DOI:** 10.3390/ijerph6020654

**Published:** 2009-02-13

**Authors:** Thomas Cochrane, Rachel C. Davey, Chris Gidlow, Graham R. Smith, Jon Fairburn, Christopher J. Armitage, Hilde Stephansen, Svetlana Speight

**Affiliations:** 1 Centre for Sport & Exercise Research, Staffordshire University, Leek Road Campus, Stoke on Trent, ST4 2DF, United Kingdom; E-mails: r.davey@staffs.ac.uk (R.C.D.); c.gidlow@staffs.ac.uk (C.G.); 2 Institute for Environment and Sustainability Research, Staffordshire University, College Road, Stoke on Trent, ST4 2DE, United Kingdom; E-mails: g.r.smith@staffs.ac.uk (G.R.S.); jon.fairburn@staffs.ac.uk (J.F.); 3 Department of Psychology, Sheffield University, Sheffield, S10 2TP, United Kingdom; E-mail: c.j.armitage@sheffield.ac.uk; 4 NatCen, National Centre for Social Research, 35 Northampton Square, London EC1V 0AX, United Kingdom; E-mails: h.stephansen@gold.ac.uk (H.S.); s.speight@natcen.ac.uk (S.S.)

**Keywords:** Physical activity, public health, environment, multi-level analysis, beliefs, intentions, perceptions of neighbourhood, area level influences

## Abstract

Reducing population physical inactivity has been declared a global public health priority. We report a detailed multi-level analysis of small area indices and individual factors as correlates of physical activity in deprived urban areas. Multi-level regression analysis was used to investigate environmental and individual correlates of physical activity. Nine individual factors were retained in the overall model, two related to individual intentions or beliefs, three to access to shops, work or fast food outlets and two to weather; age and gender being the other two. Four area level indices related to: traffic, road casualties, criminal damage and access to green space were important in explaining variation in physical activity.

## 1. Introduction

Physical inactivity is linked to poorer health [1–3]. A number of influential reports advocate a significant role for increasing population levels of physical activity to address the growing burden of non-communicable diseases, linked to lifestyle [4–7]. Notwithstanding compelling evidence and these calls for action, population levels of physical inactivity remain relatively high in most nations. For example, approximately two-thirds of men and three-quarters of women in the UK are considered not physically active enough to protect their health [5]. Moreover, this propensity to be inactive appears to begin early in life and to increase throughout the lifespan [8]. A better understanding of the determinants of physical activity is important to support the development of public health programmes aimed at increasing population levels of physical activity participation.

Most studies that have addressed this issue to date have focused on individual characteristics or circumstances such as age, gender, education, occupation, socioeconomic status and self-efficacy in order to develop interventions aimed at improving knowledge and the motivation to alter *individual* lifestyle choices in a particular setting. A recent systematic review of the effectiveness of public health interventions for increasing physical activity [9] identified common attributes of successful interventions but concluded that the evidence base for policy recommendations in the UK remains sparse. Virtually all of the interventions considered in this review targeted the individual and were limited to a specific setting. It was conceded by the report’s authors that such intervention, at its very best, will have a limited impact on population physical activity and that research on and evaluation of population-based approaches is needed.

There is growing recognition that a sedentary lifestyle is being driven, at least in part, by environmental factors that affect individuals’ physical activity choices and health behaviours. In other words, the environments in which we live, and with which we interact, have become ones that encourage lifestyle choices that decrease physical activity. A number of recent reviews signal an evolutionary shift away from individually orientated theories to broader, more environmentally based approaches for understanding and altering the determinants of population physical activity [10–13].

Neighbourhood environments may either encourage or discourage physical activity [14] and a number of theoretical models embracing environmental factors as correlates of physical activity have been proposed [15–19]. Environmental factors that have been linked to physical activity include: proximity of facilities and spaces and aesthetics [20,21], social capital [22,23], perceived safety [24,25], neighbourhood design, land use mix, transport and traffic [26], crime [27] and weather [28]. However, empirical evidence of a direct association between environmental characteristics and physical activity remains limited. What is more, most of the studies highlighted above suffer from two further limitations. Firstly, in many cases the geographical scale of the area of interest is too large to capture the detail of the interaction between individuals and their immediate environment with loss of sensitivity to detect association. Secondly, few studies have used multi-level analyses, where effects of area level factors (the higher level) on physical activity may be estimated simultaneously with individual level (the lower level) correlates.

Researchers in the United States [29,30] and in Australia [31,32] have begun to explore the importance of the ‘small’ neighbourhood scale (down to 400 m buffer around respondents’ homes in some instances). The Neighborhood Quality of Life Study (NQLS) [30] and the Physical Activity in Localities and Community Environments (PLACE) study [32] were similar in concept to the research reported here, where the primary interest is in the relationship between the neighbourhood environment and health-related behaviours such as physical activity and eating habits. The focus of these two related studies was on active transport and neighbourhood walkability defined in terms of dwelling density, connectivity, land use mix and net retail area. Our focus here is on all physical activity taken outside of the work environment (which includes active transport, garden and domestic and leisure activity but excludes physical activity in the work setting) and we extend the range of environmental factors considered, in particular, including physical activity spaces and facilities, green space and weather. We are not aware of any such studies that have been carried out in urban settings in the UK and that have included simultaneous consideration of weather (an important consideration when the prevailing weather is wet or overcast) and including all components of the available network (paths, short-cuts, cycle routes, streets and roads).

Two recent studies [33,34] have used multilevel approaches to examine the association between neighbourhood environment and physical activity. Both studies demonstrated that neighbourhood characteristics are associated with physical activity, both positively and negatively. In the Netherlands (Eindhoven), van Lenthe *et al.* [33] found that those living in more socio-economically disadvantaged areas were more likely to cycle or walk to the shops or work but were less likely to walk, cycle, garden or participate in sport in leisure time than those living in the least disadvantaged areas. In Australia (Melbourne), Kavanagh *et al.* [34] showed significant area level differences in walking, cycling and swimming. Neither of these studies, however, collected sufficient area level detail to be able to offer plausible explanations of environmental characteristics that contribute to the observed variation in physical activity. Kavanagh *et al.* go on to suggest that future research should collect detailed environmental data in order to identify key characteristics that could guide urban design to promote greater population levels of physical activity. Added to this, van Lenthe *et al.* highlight the importance of individual psycho-social characteristics such as attitudes, self-efficacy and stages of readiness to change and advocate further research that collects environmental characteristics simultaneously with such individual psycho-social factors.

In this paper, we report a detailed multi-level analysis of the role of small area indices and individual factors as correlates of physical activity across a range of areas with different degrees of socio-economic disadvantage. The research provides a detailed mapping of the urban environment down to small local area level and evaluates the relationship between environmental characteristics, individual characteristics (including socio-demographic, psycho-social and perceptions of neighbourhood information) and physical activity behaviours using multi-level analysis.

## 2. Methods

### 2.1. Settings

The study was conducted in Stoke on Trent, a mid-sized conurbation (population ∼ 240,000) located in the West Midlands region of England, UK. The geographical unit selected for the study was the Lower Level Super Output Area (LSOA). This is the smallest unit for which population census data are made available in the UK. The choices of areal unit and aggregation are themselves important considerations [35–37], firstly, because of the potential for loss of sensitivity to detect association and, secondly, because different environmental factors may operate over different areas. For example, residents may be prepared to walk longer distances in leisure time to visit a park but would not be prepared to walk the same distance to shops or to work on a more frequent basis. In England, there are 32,482 LSOAs, which have an average population of 1,500 and a minimum population of 1,000 people. Stoke on Trent has 160 LSOAs. Each LSOA is made up of 4–6 output areas (OA). Thus, within this study we have been able to examine two levels of geography (LSOA and OA) in order to investigate area level variation both within and between LSOAs.

After ranking the areas into deciles of deprivation based on the English Indices of Multiple Deprivation 2004 [38] and exclusions for areas adjacent to the city boundaries (no geographical data was available beyond the boundary) and areas undergoing significant redevelopment, ten LSOAs were selected randomly in matched non-adjacent pairs from the highest 6 deciles of deprivation (i.e. more deprived areas). The latter area sampling condition was included to allow for the possibility of evaluating effects of environmental change on population physical activity in a future cluster randomised controlled trial. It should be noted that over 90% of the population of Stoke on Trent lives within these 6 deciles of greatest deprivation.

### 2.2. Sample Population

The sample for this cross-sectional survey was a random probability sample of addresses in the selected LSOAs. A sample size requirement of 600, with approximately 60 in each area, was calculated to provide adequate precision (to within 5% with 95% confidence for a total target population ∼15,000) for target population proportion estimates. Given that there were 51 output areas (clusters) included in the study, it was estimated that this sample would allow effect sizes of 0.3 or above to be detected, assuming statistical power of 0.8, a false positive rate of 0.05, a mean of 12 respondents per cluster and an inter-cluster correlation of 0.05 [39]. The small-user version of the Postcode Address File for England was used as the sampling frame. The total issued sample size was 1,700 addresses (170 addresses per LSOA). Addresses were eligible for the survey if they were residential and occupied as a main residence and if they constituted private households. If an address comprised of several dwelling units (e.g. several flats listed together under one house number), one dwelling unit was selected randomly using a Kish grid. At each eligible address, one adult (aged 16 or over) was selected as a respondent to the survey, again using a Kish grid. There was no upper age limit for survey participants.

### 2.3. Measures

#### 2.3.1. Objective Environmental Indices

Environmental indices included were: a) proximity of physical activity spaces and facilities (using both Euclidean and network distances), b) neighbourhood connectivity [26,40], c) land use mix and population density [26,41], d) mass transport provision, e) traffic, safety and crime (from local neighbourhood statistics), f) commercial outlets, including local services, retail and food [42] and g) weather. Weather measures were all calculated from objectively measured daily weather station data gathered by the local authority. Values used were weekly average rainfall (mm), ambient temperature (°C), and sunshine (Wm^−2^) for the 7 days immediately prior to the date on which the survey was completed by each respondent.

#### 2.3.2. Physical Activity

Physical activity was assessed using the International Physical Activity Questionnaire (Long version) [43], which provides estimates of weekly energy expenditure (MET minutes/week) in four activity domains – work-related, active transport, garden and domestic, and leisure. For the purposes of this investigation, we focus on activity outside of the work domain (sum of active transport, garden and domestic, and leisure) since this was considered most likely to be associated with the participants’ residential area (i.e. work activity was undertaken, in the main, away from the respondent’s residential area).

#### 2.3.3. Individual Measures

Individual measures included: a) socio-demographic details (gender, age, ethnicity, socio-economic classification [44], education level, household tenure and income), b) perceptions of the built neighbourhood [45, adapted for use in a UK context], c) social capital [46], d) social support (friends and family) and e) beliefs about physical activity behaviour (self-efficacy, intentions, attitudes, subjective norms) [47]. As far as possible we have retained the original questions in the validated questionnaires used but have changed some of the wording to reflect the appropriate terms in the UK. For example, “row houses” has been replaced by “terraced houses”, “apartments or condos” have been replaced by “flats or apartment blocks”, “Stores” has been replaced by “Shops”. Questionnaire contents were as follows: perceptions of neighbourhood - 40 items (17 scored on a 5-point ordinal scale and 23 scored on a 4-point ordinal scale expressing degree of agreement with a given statement); social capital - 12 items (8 scored on a 4-point ordinal scale expressing degree of agreement with a given statement and 4 dichotomous items); social support – 7 items scored on a 3-point ordinal scale expressing degree of agreement with a given statement; attitudes, intentions and beliefs – 8 items scored on a 7-point ordinal scale expressing degree of agreement with a given statement, anchored by polar alternatives.

### 2.4. Procedures

The survey was carried out independently by the National Centre for Social Research (NatCen) between May and October 2007 and comprised a 45-minute interview, administered using computer assisted personal interviewing, and measurement of height and weight. In order to validate the physical activity questionnaire, respondents in a randomly selected sub-sample (target ∼ 100 participants) were also asked whether they would be willing to wear an accelerometer (Actigraph GT1M, USA) for 7-days to monitor their physical activity. If consent for the latter was given, the respondent was then contacted by the research team, fitted with an accelerometer and asked to return the device after one week using a prepaid return envelope. All accelerometers were fitted as soon as was feasible after completion of the questionnaire, mostly within two days but up to a maximum of 5 days. Correlation between self-reported activity (total activity in MET minutes/week) and accelerometer measured activity (total activity count/ total effective recording period in minutes) was considered moderate to good at 0.57 (n=109).

Following advance letters to sampled addresses, interviewers made contact with respondents by personal visit and were required to make a minimum of four calls at different times of the day and on different days of the week before recording a ‘non-contact’ outcome. Once contact with the selected person was made, the interviewer introduced the survey, presented the information leaflet, described procedures to insure confidentiality of data usage, answered any questions and sought respondents’ consent to take part in the study. Full details of the interview procedures are contained in the survey technical report [48]. The study was approved by the Staffordshire University Research Ethics Committee.

Geographical Information System (GIS) analysis has been used to derive area level indices, of which over 1,200 have been evaluated. In addition, because of wide variation within LSOAs, we have re-aggregated data and calculated indices for a total of 51 output areas (∼125 households) contained in the study. Various buffer distances, from 200 m up to 1 km, have been used to explore this important aspect of sensitivity of the GIS analysis. Full details of the procedures used to derive these indices are beyond the scope of this paper but are available in the GIS Technical Report from the project [49], also provided as a supplement to this paper.

### 2.5. Data Analysis

The purpose of our analysis was to determine the combination of individual and small area level indices that best explained the observed variation in population physical activity outside of the work domain using a multi-level linear regression model. Inspection of the distribution of the physical activity outside work data demonstrated significant positive skew that was transformed to a normal distribution using square root transformation. Thus, all analyses reported have used the square root transformed data. A multi-stage strategy was used to develop our explanatory model, firstly, establishing a base set of individual factors and then examining area-level indices through their influence on these individual factors.

Individual item responses were examined separately and considered for inclusion in a multiple linear regression sub- model if they demonstrated a linear relationship with the outcome measure (inspection of scatter plots (continuous variables), error bar plots (ordinal variables) and significant correlation, p<0.05) with good coverage of the range and no outliers. Separate multiple linear regression models were developed for each measure (demographics, neighbourhood perceptions, social capital, social support, attitudes, intentions and beliefs). Weather was also considered at the individual level in this analysis, using average weekly rainfall, sunshine and temperature during the week for which the physical activity questionnaire was reported by each participant. All retained variables in these separate sub-models were then entered in a final stage linear regression analysis to establish a base set of key individual predictors.

A similar process was used to establish potential predictor variables at area level. Initially, proposed indices for inclusion in the models were those that demonstrated significant correlation (p<0.05) with mean activity within the area. This process yielded 22 possible correlates (18 negative and 4 positive) in this analysis. For each potential correlate, scatter plots of mean physical activity outside work (transformed) against each index were then examined carefully. Those that demonstrated linearity, good coverage of range and no outliers were included for further investigation.

The final stage in the analysis was to combine the individual level and area level indices to develop the overall multi-level prediction model. The level-1 model was of the form:

(1) $${\text{Y}}_{\text{ij}}=\beta_{0\text{j}}\;+\sum_{q=1}^{Q}\beta_{\text{qj}}{\text{X}}_{\text{qij}}+{\text{r}}_{\text{ij}}$$

where

Y_ij_: is the outcome measure of interest for participant i in area j
β_qj_: (q=0, 1,.. ..,Q) are level-1 intercept and coefficients
X_qij_: is the level-1 predictor q for participant i in area j
r_ij_: is the random effect at level-1

Level-2 (area) indices were modelled through their influence on the level-1 factors, wherein each level-1 coefficient β_qj_ was considered an outcome variable in the level-2 model:

(2) $${\text{β}}_{\text{qj}}=\gamma_{\text{q}0}+\sum_{s=1}^{S}\;\gamma_{\text{qs}}{\text{W}}_{\text{sj}}+{\text{u}}_{\text{qj}}$$

where

γ_qs_: (s=0,1,.. ...,S) are the level-2 intercept and coefficients
W_sj_: is the level-2 predictor
u_qj_: is the random effect on level-1 coefficient q at level-2

Equation (2) allows for each level-1 coefficient to be modelled either in fixed, non-randomly varying, randomly varying or a combination of randomly and non-randomly varying forms. Building of the multi-level model was, correspondingly, rather complex. Our approach, for each retained area level index separately in the first instance, was to use the non-randomly varying form of the equation (2) (u_qj_ set to 0) as a starting iteration. Level-2 coefficients, γ_qs_, making a significant (p<.05) explanatory contribution to the variation in the model were retained and further iterations were carried out adding a randomly varying component (u_qj_) to test if this significantly improved the model fit. Finally, these separate level-2 models were combined into an overall best-fit multivariate (at both level-1 and level-2) multi-level linear regression model, again retaining only those coefficients making a significant (p<0.05) contribution to the overall model fit. All analyses were carried out using HLM-6 (Scientific Software International, Lincolnwood, IL, USA) using level-1 and level-2 data files created with SPSS Version 15 (SPSS Inc, Chicago, IL, USA).

## 3. Results

### 3.1. Response to Survey

Of the 1,700 addresses sampled, 1,545 were considered eligible and, from these, 761 (49%) productive interviews were obtained. The response rate of 49% was a few percentage points lower than what might be expected for a survey of this type (given the topics covered and duration). Clustering of the sampling over relatively small areas in the more deprived parts of Stoke on Trent and the short time frame for data gathering were thought to be the main factors contributing to the slightly lower response rate.

### 3.2. Demographics

Just over half (55%) of the sample were females and the ethnic mix of the population was predominantly white Caucasian (93%), reflecting the ethnic mix of the population of Stoke on Trent (Table 1). The majority of the population was overweight (65%), of low educational attainment (72%) and modest income. The sample achieved good coverage of the age ranges and socio-economic status.

### 3.3. Physical Activity Levels and Intentions

Key findings from the survey in relation to participation in physical activity, and intentions to participate in physical activity to levels recommended, in the future by gender are summarised in Table 2. Overall, physical activity participation was dominated by that associated with work (43.4%) and garden and domestic activity (32.2%) with active transport and leisure activity each accounting for ∼ 12% of total activity (Table 2a). The majority of those surveyed (65%) indicated that they did not intend to participate in recommended levels of physical activity in the future, while 16% considered that they already met recommended levels (Table 2b).

The sample size per output area ranged from 5 to 26 with a mean of 15 (Table 3). Given the imbalance in the number per cluster (output area) in our sample, it was important to check the potential for small sample bias in the multi-level analysis by comparing the weighted mean for the transformed activity outside work against the arithmetic mean over the 51 output areas [50]. These values were 58.65 and 58.01 respectively. Thus fixed effects estimates in this analysis were considered unlikely to be biased by a small sample effect.

### 3.4. Physical Activity Outside of Work and Associated Individual Factors

Table 3 also shows physical activity outside of work by census output area within the 10 study areas. It can be seen from this that reported physical activity outside of work varied widely both within and between areas. This variation was associated, at the population level, with a number of factors, which are summarised in Table 4. These are the 11 factors retained in the explanatory model developed from the 67 individual survey items plus age, gender and the three weather items.

It can be seen from Table 4 that individual beliefs and self-confidence in ability to participate in physical activity were generally positive across all areas (mean score in the range 4.1 to 5.3 on a 1–7 scale) whereas intention to participate in physical activity in the future was low (∼ 2 on the 1–7 scale). Access to fast food outlets, supermarkets or convenience stores was generally good across all areas (mean scores ∼ 2 on a 5-point scale ranging from 1 - < 5 minutes to 5 > 30 minutes), while access to work or place of study was not so convenient (mean score 4.1 to 4.7 on the same 5-point scale). The majority of respondents in all areas agreed that there were several shops within easy walking distance of their home (mean score 2.7 to 3.6 on a 4-point ordinal scale ranging from 1 - strongly disagree to 4 - strongly agree).

### 3.5. Explanatory Models

Multiple linear regression models for perceptions of neighbourhood, social capital, beliefs about physical activity and all retained individual-level factors are shown in Table 5.

Five items were retained from the perceptions of the built environment questionnaire. Closer walking proximity of convenience stores, other shops, fast food restaurants and work or place of study were all associated with higher reported levels of physical activity outside of work, as was attractiveness of buildings. In relation to social capital items, close proximity to a supermarket was the strongest correlate but neighbours looking after each other and participation in neighbourhood groups were also significant. Individual intention to participate in physical activity at the recommended level (moderate physical activity for 30 minutes or more at least 5 times a week) was the dominant factor amongst the beliefs, intentions and attitudes items, although belief in ability, confidence in ability and intention to participate in physical activity in the future each made significant explanatory contributions in the model.

**Figure 1.**
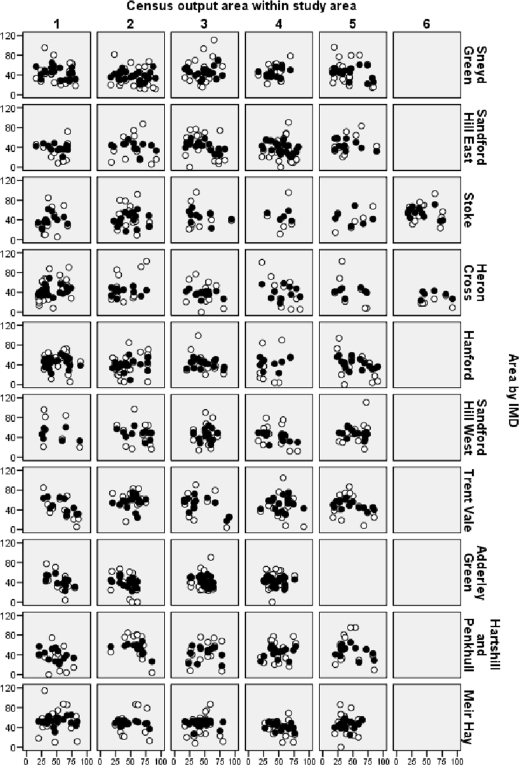
Activity outside work (transformed) vs age (years) within the 51 study areas. Key: ○ - actual data, • - multi-level fit model.

When all factors were included in the combined models, eleven items were retained. Overall, these factors explained about 22% of the variability observed in reported activity outside work, supporting the potential importance of the environment in influencing physical activity. Individual beliefs or intentions made the dominant explanatory contribution. When considered separately these items explained ∼17% of the variation whilst neighbourhood perceptions and social capital items, considered on their own, explained 7.5% and 7.2% respectively. Figure 1 shows the overall fit of the model to the data while the combined multi-level model is summarised in Table 6 (fixed effects) and Table 7 (random effects). It can be seen from Figure 1 that the model captures the general trend in physical activity behaviour quite well in most areas but significant variation remains unexplained. This figure also highlights the variation both within areas and between areas. Finally, it flags up the complex relationship between physical activity, environment and age, with some areas showing a trend towards increased activity with age, some showing a trend towards decreased activity with age and others showing an inverted ‘U’ relationship.

When environmental factors were added to the model, two individual level factors, proximity of a supermarket and intention to participate in physical activity in the future, were no longer retained in the model, indicating, perhaps, that these two items were dominated by environmental characteristics. Four area-level factors made significant explanatory contributions in the model. Length of road with moderate traffic levels (within an 800m buffer area around the output area) made an independent positive contribution to explaining observed variation in physical activity, whilst count per km of road of casualties involving public transport and count per head of population reporting criminal damage made independent negative contributions. Further significant environmental contributions were observed through their influence on individual factors. Count per km of road of casualties involving public transport made a positive contribution to physical activity through the item related to accessibility of local shops and a negative contribution through the belief in ability to participate in physical activity item. Accessibility to recreational green space made a negative contribution to physical activity during sunnier weather and a positive contribution in wetter weather. Criminal damage was associated with increased activity during sunnier weather.

## 4. Discussion

### 4.1. Levels of Physical Activity

Reported levels of physical activity in this sample population were low and dominated by work and garden and domestic activity for both males and females, 78% and 73% respectively. Considering the mean values reported in Table 2 (formal significance testing for each comparison was performed using independent samples t-test on the transformed data), males were more active in the work domain than females (p<0.001), less active in garden and domestic activities (p=0.003) and not significantly different in either leisure or active transport activity. Closer inspection of Table 2(a) reveals that median levels of physical activity in all domains were much lower than the respective means, indicating that the data distributions were positively skewed and that large proportions of both males and females reported very low levels of physical activity in all domains. In fact, median levels of activity in work for both males and females and in leisure for females were zero. These findings are in keeping with the headline results from the Active People Survey, the largest ever survey of sport and active recreation in Europe [51], which showed the proportion of those participating in regular moderate levels of physical activity in Stoke on Trent to be among the lowest in England at 15.8% (this survey considered those meeting the level of 30 minutes moderate activity on at least 3 days). If regular participation in moderate amounts of physical activity is considered important for the maintenance of good health, then these low population levels of physical activity must be a public health concern.

### 4.2. Intentions, Beliefs and Attitudes towards Physical Activity

Of even more concern, perhaps, where the political climate favours the importance of individual choice, is the data reported in Table 2(b) which shows that ∼65% of the population do not intend to maintain regular moderate physical activity to recommended levels in the future. This finding questions both the impact of public health messages and public health policy relating to physical inactivity. Sixteen percent of the population considered themselves already active to recommended levels while a further 19% indicated some intention to be more active. The latter indicates at least some scope for improvement, though this may need to be supported by targeted intervention.

### 4.3. Influence of the Neighbourhood Environment

On a more positive note, this research confirms the importance of the neighbourhood environment in determining levels of physical activity participation, both negatively and positively. Of the nine individual factors retained in the overall model (Table 6), two were related to individual intentions or beliefs, three were related to access to shops, work or fast food outlets and two were related to weather; age and gender being the other two. These, in turn, were influenced by four indices calculated at area level, one related to traffic, one related to road casualties involving public transport, one related to criminal damage and one related to access to green space.

The data in Table 3 demonstrate that the variation in mean physical activity outside of work by OA within each LSOA was marked, as was the variation within each OA. The implication of this finding is that analysis based on the LSOA as the area of interest would have resulted in a loss of sensitivity to detect correlation with physical activity (overall variation to be explained was ∼350 MET minutes/week, compared with the variation of ∼ 2,300 MET minutes/week when considered at the OA level). Furthermore, when considering how individuals live and interact with their environment, planners and policy makers may need to examine local geography to a lower level than is commonly the case, particularly in relation to designing in features that will encourage physical activity.

Of further interest in this analysis, are the ways in which the area level factors appear to operate, or interact, with individual level factors. For example, the background level of physical activity outside work in each area was influenced positively by moderate levels of road traffic and negatively by road traffic accidents (in a bus in this instance) and criminal damage. The positive influence of moderate levels of road traffic may simply be a reflection that areas that have more traffic traveling at moderate speeds have more activity going on generally. The number of casualties involving public transport had a negative influence on background level of activity in an area and on individual’s belief in their ability to participate in physical activity and a positive influence through walking access to local shops.

Overall, access to destinations was positively associated with activity outside of work, in agreement with similar findings reported elsewhere [29,52,53], though it is difficult to compare these studies directly because of differences in the areas studied, the country of study and the measures and models used. In contrast with the results reported by Leslie *et al.* [54], women in our sample were not less active than men in leisure activity. Moreover, women were more active (outside of work) than men, reporting higher levels of active transport and garden and domestic activity; reinforcing the need for context specificity when considering physical activity, as suggested by Giles-Corti *et al.* [55].

Age was positively associated with physical activity outside work, which is in contrast with the general belief that physical activity declines steadily with age from early adulthood through to old age and with the finding of Cerin *et al.* [53] that age was negatively associated with active transport. In our best fit model, age was the only variable that supported the inclusion of a random effect, reflecting, perhaps, a more complex interaction with age than the simple linear relationship expected.

Five perceptions of neighbourhood items, when these were considered alone, explained about 7.5% of the observed variation in physical activity, Table 5. Similarly, five items from the social capital module, when considered alone, explained about 7.2% of the observed variation in physical activity. These are significant influences and, collectively, support the importance of destinations that matter i.e. shops, stores, food outlets, work or study sites, and venues that allow groups to meet or to interact socially, all within close proximity to the place of residence. There is an important message here to planners and policymakers considering residential development or neighbourhood renewal to design physical activity in by including some or all of these features rather than to design it out by neglecting to include them. Although our studies are not directly comparable, Frank *et al.* [30] have reported a similar degree of influence for objectively measured indices of walkability on active transport in their US study and Cerin *et al.* [53] report similar findings from Australia.

Looking at the model as a whole, the greatest explanatory contribution was made by individual intentions and beliefs, indicating that a large proportion of physical activity behaviour may be mediated by cognitive factors. However, a significant proportion of the explained variance remained over and above that attributable to these factors, supporting the postulate that some environmental influences may be unmediated by cognitive factors. For a discussion of this issue, see Kremers *et al.* [56]

Access to recreational green space ≥ 2 hectares was influenced by the weather but the direction of this influence ran counter to expectations. Greater population access to green space had a negative influence in sunnier weather and a positive influence during wetter weather. It was not feasible, in this analysis, to gather reliable data on the functionality of available green space or to gather detail of what activities were undertaken where. One explanation for the pattern observed here could be that those with poor access to functional green space were prepared to walk further to access green space outside their area in sunnier weather but accessed more local green space when the weather was wet. Similar findings have been reported elsewhere [57]. In a recent review of associations between physical activity and access to parks and recreation settings, Kaczynski and Henderson [58] reported that, though most of the articles reviewed reported some significant positive relationships between physical activity and parks and recreational settings, a significant number of studies reported mixed or no associations, prompting these authors to conclude that, because of the ubiquity of parks and recreation spaces, their potential contribution to active living merits further exploration. Our findings support this potential need for more effective use of available green space.

Our research is consistent with other evidence in supporting the importance of the built environment in determining population levels of physical activity. Through the use of multi-level modelling, it extends our understanding of the importance, and the complexity, of the interaction between individual behaviours and the living environment. In terms of implications for urban planning, the study underscores the need to build walking access to shops, work and other services into the neighbourhood environment. In general, proximity of the population to potentially usable green space was good in most areas. However, actual use may not have been widespread since reported levels of physical activity in leisure time were low. This accessible green space represents a potential resource that could be better utilised, with resulting benefits to health and perceptions of the environment [59–61].

### 4.4. Perceptions of Neighbourhood

When examined separately, perceptions of neighbourhood and social capital items were associated with physical activity (Table 5) but their effect may be mediated through individual intention or belief, since many of these items were not retained when individual intentions and beliefs were included in the regression analyses. This observation merits further research. Change in public health behaviour is likely to be mediated by change in individual intention [62]. Thus, the specific research need is to identify environmental and/or policy changes that are likely to lead to a positive change in intention to be more physically active. This, in turn, would allow well-designed research studies to be developed to test whether such changes do indeed lead to reduction of current high levels of inactivity.

### 4.5. Limitations

Although our research confirms associations between neighbourhood environment and physical activity, a number of limitations need to be considered alongside our interpretation of these findings. Chief among these must be sample size. Overall, we obtained good survey data from a representative random sample of 761 respondents from 10 neighbourhoods, made up of 51 smaller neighbourhood areas. On the basis of this, estimates of population proportions for Stoke on Trent are likely to be reliable but uncertainty about how well the sub-sample at individual output area level captures the mean level and variation of physical activity must be accepted. We are confident that, for this population in this setting, environmental factors explained some of the variation in observed physical activity. How much of this may be generalised to other populations in other settings can only be tested by further research to see if effects observed here can be replicated. A further limitation of sample size was that the power to detect effects in multi-level analyses was limited to moderate effect sizes only. Thus, there could be more subtle area-level effects not detected here. One way round this would be to calculate each index in relation to individual household and carry out multiple linear regression analysis for all 761 respondents.

A further limitation of this research relates to the method of measurement of physical activity, in this case the long version of the International Physical Activity Questionnaire. This is a self-report, 7-day re-call instrument that allows estimates of physical activity in MET minutes/week to be obtained in four domains. Unfortunately, it makes no provision for recording where the activity takes place or of its timing. For this analysis, we have assumed that individual activity outside of the work domain will be undertaken, at least in part, around the home environment. However, it is also true that some of the activity outside work may well take place elsewhere, which adds unknown variability to the outcome measure and limits the explanatory power of the model.

Finally, the data gathered here were cross-sectional in nature. Thus it is not feasible to attribute causality. However, this mapping and modeling study has been conducted to provide baseline data with which to develop and evaluate targeted environmental interventions designed to increase the proportion of the community that is sufficiently active to benefit health.

## 5. Conclusions

This study supports the proposal that residential neighbourhood down to small area level may be important in determining physical activity. Reported levels of physical activity were low so it may be suggested that local environments were generally not supportive of regular physical activity. This point is amplified by the finding of a population intent on maintaining low levels of physical activity. It is reasonable to conclude from these observations that environmental change on its own is unlikely to alter physical activity behaviour unless accompanied by support to change individual beliefs, self-efficacy and intention to be physically active. Based on the multi-level analysis reported here, key conclusions for urban planners in terms of promoting physical activity would be to design in walking access (ideally within 1–5 minutes) to work, shops and local services and to provide for better public use of green space that was widely available throughout the areas sampled. Further, traffic calming and re-routing should be considered to reduce the incidence of urban road accidents.

## Figures and Tables

**Table 1.** Demographic characteristics of the sample.

Characteristic	Category	N	%
Gender	Male	343	45.1
	Female	418	54.9
Age group	15–24	76	10
	25–44	276	36.3
	45–64	235	30.9
	65+	174	22.9
Ethnicity	Caucasian	709	93.2
	Mixed	12	1.6
	Asian	22	2.9
	Black	5	0.7
	Other	13	1.7
Weight (BMI) category	Underweight	10	1.3
	Acceptable	256	33.6
	Overweight	283	37.2
	Obese	212	27.9
Education	None	3	0.4
	Secondary school (< = age 16)	547	71.9
	Sixth form college (age 17–19)	110	14.5
	Higher education	101	13.3
Number in household	2 or less	510	67
	3–5	240	31.5
	>5	11	1.4
Socio-economic status	Managerial and professional	182	23.9
	Intermediate	72	9.5
	Small employer or own account	41	5.4
	Lower supervisory and technical	116	15.2
	Semi-routine	312	41
	Not applicable	38	5
Annual household income^a^	< £10,000	178	23.5
	£10,000 - < £20,000	167	22
	£20,000 - < £30,000	199	26
	>= £40,000	72	9.5
	Not answered	145	19

a £, GB pound ≈ 1.25 euro (€), 1.89 UD dollar ($) during study period

**Table 2.** Summary of: (a) participation in physical activity and (b) future intention to participate to recommended levels.

(a)						
Activity domain^a^	Male (N=343)			Female (N=418)		
	Sum	Mean	Median	Sum	Mean	Median
Work	865643	2524	0	593711	1420	0
Active transport	153342	447	165	250807	600	198
Garden & domestic	434798	1268	660	648052	1550	1028
Leisure	207274	604	132	209556	501	0

(b)			
Intention^b^	Male	Female	Total
Yes	60	86	146
	7.9%	11.3%	19.2%
No	221	272	493
	29.0%	35.7%	64.8%
Already active	62	60	122
	8.1%	7.9%	16.0%

a All activity reported in MET minutes/week.

b To participate in moderate physical activity for at least 30 minutes 5 times a week.

Sum = sum of all activity in that domain

**Table 3.** Summary of physical activity outside of work (MET minutes/week) by census output area within study areas^a^.

Area^b^	OA^c^	1	2	3	4	5	6	Overall
Sneyd Green	Mean	2412	1700	3237	2496	2847		2472
	SD	2252	1625	3231	1496	2565		2305
	N	20	26	18	16	19		99
Sandford Hill	Mean	1345	2236	2391	1696	2558		2021
East	SD	1459	2525	2107	1975	2067		2041
	N	12	12	20	24	11		79
Stoke	Mean	1966	2730	2657	2630	1908	3530	2653
	SD	2290	2293	3019	3686	1593	2222	2428
	N	12	18	9	5	6	13	63
Heron Cross	Mean	2767	3423	1983	2615	2788	484	2526
	SD	2389	3595	1902	3179	3805	373	2766
	N	22	12	12	10	7	6	69
Hanford	Mean	2499	1922	2286	2272	2163		2220
	SD	1563	1903	2235	3179	2306		2104
	N	20	21	17	9	16		83
Sandford Hill	Mean	3923	2695	2568	2429	2978		2810
West	SD	3653	2769	2337	1998	3035		2633
	N	7	11	14	14	13		59
Trent Vale	Mean	2229	4097	3040	2965	2785		3102
	SD	2194	1648	2564	2623	2301		2299
	N	11	18	12	18	16		75
Adderley	Mean	2002	1898	2061	2081			2024
Green	SD	1896	1556	1828	1344			1594
	N	11	16	22	26			75
Hartshill & Penkhull	Mean	1533	4006	2344	2649	3255		2734
	SD	1541	2582	2019	1972	3109		2368
	N	13	12	14	17	14		70
Meir Hay	Mean	3574	2875	2284	1631	2659		2608
	SD	3139	2487	2007	1223	1979		2301
	N	19	14	20	17	19		89

a All activity reported in MET minutes/week.

b Study area, arranged in order of Index of Multiple Deprivation, most deprived first.

c OA – census output area within selected area. SD – standard deviation. N – number of respondents.

**Table 4.** Summary statistics for each factor retained in the level-1 (individual) model.

Factors retained in Level 1 (individual) model^a^	Mean	SD	Range	
Belief in ability to participate	4.8	0.38	4.1	5.3
Intent to participate in recommended PA	2	0.09	1.8	3.1
How confident will be able to participate	4.6	0.31	4.1	5.1
Age of respondent (years)	48.4	2.27	46	52
Sex of respondent Female	42	9	29	59
Male	34	5	26	40
Sunshine (W/m2)	175.5	7.16	165	189
Walking distance to fast food restaurant	1.9	0.2	1.6	2.2
How easy to get to supermarket	1.4	0.16	1.2	1.7
Several shops within easy walking distance	3.2	0.24	2.7	3.6
Walking distance work/place of study	4.5	0.2	4.1	4.7
Rainfall (mm)	3.1	0.69	2.4	4.2

a Arranged in order of relative explanatory contribution to the model, most significant factor first. SD = standard deviation.

**Table 5.** Summary of the multiple linear regression model fits to individual constituent questionnaire measures.

Outcome measure: physical activity outside work (square root transformed)					
Model^a^	B^b^	SE^c^	Beta^d^	Sig^e^	Adj^f^
Perceptions of neighbourhood items					R^2^
(Constant)	49.27	5.54		<0.001	0.075
Walking distance to local convenience store	−2.47	0.96	−0.11	0.010	
Several shops within easy walking distance	2.6	0.91	0.11	0.004	
Walking distance to work/place of study	−2.13	0.74	−0.10	0.004	
Walking distance to fast food restaurant	−1.98	0.81	−0.1	0.014	
Attractive buildings or homes in neighbourhood	1.99	0.86	0.08	0.022	
Social capital items					
(Constant)	64.15	3.721		<0.001	0.072
How easy to get to supermarket	−5.92	1.21	−0.17	<0.001	
Whether participates in groups/organisations	−4.09	1.72	−0.09	0.018	
Neighbours look after each other	−2.63	0.96	−0.1	0.007	
Tenants /residents group	9.192	3.89	0.09	0.018	
Parent/teacher or school associations	9.806	4.27	0.08	0.022	
Beliefs about physical activity items^g^					
(Constant)	9.536	3.130		0.002	0.172
Belief in ability to participate	1.699	0.549	0.171	0.002	
Intent to participate in recommended PA	8.584	1.284	0.221	<0.001	
Whether intends to participate in future	1.109	0.457	0.113	0.015	
How confident will be able to participate	1.199	0.558	0.117	0.032	
All retained predictors					
(Constant)	−3.14	7.8		0.69	0.223
Belief in ability to participate^g^	2.205	0.524	0.222	<0.001	
Intent to participate in recommended PA	8.218	1.252	0.212	<0.001	
Sunshine (W/m2)	0.054	0.019	0.097	0.005	
Walking distance fast food restaurant	−1.74	0.71	−0.08	0.014	
Sex of respondent	4.864	1.494	0.105	0.001	
How confident will be able to participate	1.466	0.525	0.143	0.005	
Age of respondent	0.147	0.047	0.118	0.002	
How easy to get to supermarket	−2.56	1.16	−0.08	0.027	
Walking distance workplace/place of study	−1.56	0.691	−0.07	0.025	
Several shops within easy walking distance	1.792	0.823	0.075	0.030	
Rainfall (mm)	−0.569	0.276	−0.07	0.040	

a Coefficients shown are those retained as significant in the final stepwise regression analysis.

b Regression coefficients.

c Standard error of regression coefficient.

d Standardised regression coefficient.

e Significance level.

f Adjusted R^2^ for the model.

g Item wording has been abbreviated for Table presentation purposes. PA - physical activity.

**Table 6.** Summary of fixed effects for the multi-level model for activity outside work.

Fixed effect		γ_qs_	Coeff^a^	SE^b^	t-ratio	Df^c^	p^d^

Individual level factors	Area level factors						
Intercept, β_0_							
	Intercept	γ_00_	24.8414	6.5750	3.778	743	<0.001
	Length of road with moderate traffic level within 800m buffer	γ_01_	1.8201	0.5897	3.086	743	0.003
	Count per km of road of casualties in a bus	γ_02_	−68.7766	21.8414	−3.149	743	0.002
	Count per head of population reported criminal damage	γ_03_	−384.7072	107.6481	−3.574	743	0.001
Gender, β_1_							
	Intercept	γ_10_	4.8255	1.2398	3.892	743	<0.001
Age, β_2_							
	Intercept	γ_20_	0.1153	0.0469	2.459	50	0.018
Walking distance to fast food restaurant, β_3_							
	Intercept	γ_30_	−1.7783	0.8195	−2.17	743	0.03
Walking distance to work/place of study, β_4_							
	Intercept	γ_40_	−1.6356	0.7598	−2.153	743	0.031
Several shops within easy walking distance, β_5_							
	Intercept	γ_50_	2.9000	0.7446	3.895	743	<0.001
	Count per km of road of casualties in a bus		16.3428	6.2158	2.629	743	0.009
Whether intends to participate in recommended PA in the future, β_6_							
	Intercept	γ_60_	1.2705	0.4093	3.104	743	0.002
Belief in ability to participate, β_7_							
	Intercept	γ_70_	2.0130	0.4502	4.471	743	<0.001
	Count per km of road of casualties in a bus	γ_71_	−12.2078	4.3585	−2.801	743	0.006
Sunshine, β_8_							
	Intercept	γ_80_	0.0565	0.0209	2.711	743	0.007
	Percentage of population < 200m of unrestricted recreational green space >= 2 hectares	γ_81_	−0.0006	0.0003	−2.028	743	0.043
	Count per head of population reported criminal damage	γ_82_	1.8775	0.5937	3.162	743	0.002
Rainfall, β_9_							
	Intercept	γ_90_	−0.6738	0.3104	−2.171	743	0.03
	Percentage of population < 200m of unrestricted recreational green space >= 2 hectares	γ_91_	0.0310	0.0106	2.93	743	0.004

a Fit coefficient.

b Standard error of fit coefficient.

c Approximate degrees of freedom.

d Significance value for fit coefficient. PA - physical activity

**Table 7.** Summary of random effects for the multi-level model for activity outside work.

Outcome measure: physical activity outside work (square root transformed)					
Random effect	SD^a^	Variance component	df^b^	Chi-squared	p^c^
Age, u_2_	0.07615	0.00580	50	73.101	0.018
Level-1, r	20.30839	412.43086			

a Standard deviation.

b Degrees of freedom.

c Significance level.
